# Concomitant osteoporotic vertebral compression fracture and infectious spondylitis in a patient with herpes zoster: A case report

**DOI:** 10.1097/MD.0000000000048493

**Published:** 2026-04-24

**Authors:** Jaeeun Lee, Chung Hun Lee

**Affiliations:** a Department of Anesthesiology and Pain Medicine, Korea University Medical Center, Guro Hospital, Seoul, Republic of Korea.

**Keywords:** ferpes zoster, infectious spondylitis, osteoporotic vertebral compression fracture, physical examination, vertebroplasty

## Abstract

**Rationale:**

The incidences of herpes zoster, osteoporotic vertebral compression fractures, and infectious spondylitis is increasing worldwide owing to the aging population. Given the potential for multiple comorbid conditions with overlapping clinical presentations, thorough history-taking and physical examination are essential for accurate diagnoses. However, the coexistence of herpes zoster, osteoporotic vertebral compression fractures, and infectious spondylitis in a single patient is exceedingly rare, making timely diagnosis even more challenging.

**Patient concerns:**

A 64-year-old woman with a medical history of hypertension, asthma, and right-sided hemiplegia secondary to a previous hemorrhagic stroke presented with painful vesicular lesions in the right T10 dermatome consistent with herpes zoster. Despite antiviral and antibiotic therapy, her pain worsened, and inflammatory markers increased significantly.

**Diagnoses:**

Physical examination revealed midline spinal tenderness and pain, exacerbated by postural changes. Magnetic resonance imaging confirmed a collapsed L3 vertebral body consistent with Kummell’s disease, along with paravertebral soft tissue changes and psoas muscle involvement, suggesting coexisting infectious spondylitis.

**Interventions:**

Although the biopsy cultures were negative, empirical intravenous cefazolin therapy was initiated. After 2 weeks of intravenous antibiotic treatment, the C-reactive protein and procalcitonin levels normalized. Given the persistent mechanical back pain and stabilized infection, a vertebroplasty was performed.

**Outcomes:**

After the vertebroplasty, the patient experienced rapid pain relief without any signs of recurrent infection. Following a 5-week course of intravenous antibiotic therapy and an additional 2 months of oral administration, the patient achieved complete clinical resolution with full remission of both infectious manifestations and zoster-associated sequelae.

**Lessons:**

This case underscores the importance of a comprehensive clinical assessment in older patients, even when a diagnosis of herpes zoster is apparent. Persistent or atypical symptoms warrant further evaluation to exclude concurrent diseases. Furthermore, vertebroplasty can be safely performed in select patients with infectious spondylitis following adequate antibiotic therapy, leading to pain reduction. A multidisciplinary, individualized approach is essential to achieve optimal outcomes in patients with complex spinal pathologies.

## 1. Introduction

The global increase in the older population has led to a higher incidence of age-related conditions, including herpes zoster (HZ), osteoporotic vertebral compression fractures (OVCFs), and infectious spondylitis.^[1-6]^ These conditions cause significant pain, severely impair quality of life, and may contribute to decreased life expectancy in severe cases. Therefore, an early diagnosis and appropriate management are essential.^[1-6]^

HZ, which primarily affects older adults, results from the reactivation of the latent varicella–zoster virus in the trigeminal or dorsal root ganglia. Diagnosis is primarily based on a characteristic rash and localized pain rather than on imaging or laboratory findings.^[7]^ The hallmark clinical features of HZ include sharp, localized pain and a unilateral vesicular rash confined to a single dermatome, which typically does not cross the midline.^[8,9]^

OVCFs commonly occur in older individuals with osteoporosis. Patients typically present with back pain that worsens with postural changes or weight-bearing activities. Patients may also report band-like pain localized to the fracture site.^[10,11]^ Physical examination findings that support OVCF diagnosis include midline spinal tenderness, pain elicited by vertebral percussion, and symptom exacerbation with spinal rotation or postural changes.^[3,12]^

Although relatively uncommon, infectious spondylitis remains a critical differential diagnosis for back pain and is typically characterized by persistent symptoms that do not improve with rest. Such pain may be associated with systemic symptoms, including fever and elevated levels of inflammatory markers such as erythrocyte sedimentation rate (ESR) and C-reactive protein (CRP), which suggest an underlying infectious etiology.^[13]^ Despite their clinical importance, reports on vertebral augmentation procedures following infectious spondylitis, such as vertebroplasty (VP) or kyphoplasty (KP), are limited and the safety and efficacy of these interventions remain poorly characterized. This knowledge gap highlights the need for further clinical observation.

Magnetic resonance imaging (MRI) and radionuclide bone scintigraphy represent gold standard imaging modalities for diagnosing OVCFs and infectious spondylitis.^[5,12]^ However, owing to their cost and accessibility, these imaging modalities are not routinely performed unless specifically indicated by physical examination or laboratory findings.^[13]^ Moreover, even when clinically indicated, imaging may be delayed if alternative causes of pain and inflammation are considered more likely, potentially hindering early diagnosis of OVCFs or infectious spondylitis.

Therefore, the physical examination remains a vital tool for clinicians to directly and promptly assess a patient’s condition, particularly when a timely diagnosis is essential. This study presents the case of a patient who was initially diagnosed and treated for HZ based solely on the presence of severe thoracic pain and a vesicular rash. However, following a detailed clinical history, focused physical examination, and subsequent MRI, the patient was found to have coexisting OVCF and infectious spondylitis. This case underscores the critical importance of a thorough clinical assessment, including detailed history and physical examination, to ensure accurate diagnosis and appropriate management.

## 2. Case presentation

This study was approved by the Institutional Review Board of Korea University Medical Center, Guro Hospital, Seoul, Republic of Korea (2025GR0119) on February 27, 2025.

**Figure 1.**
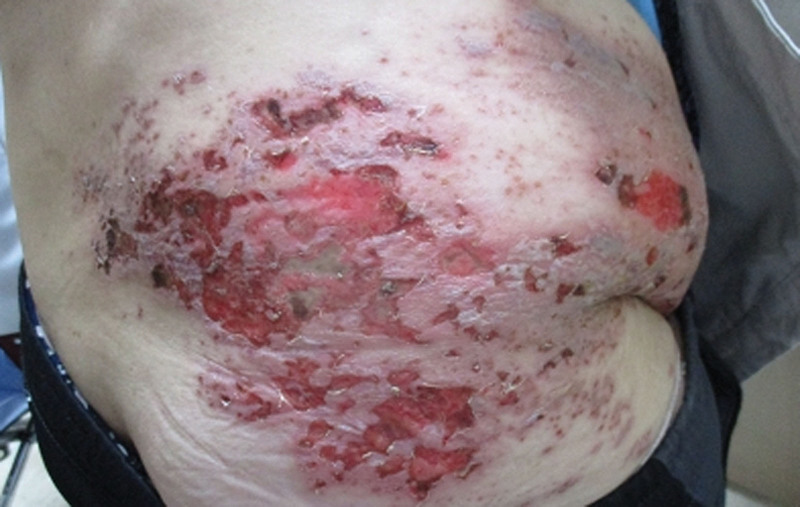
Photograph obtained at the initial presentation (1 week after onset of herpes zoster infection), showing vesicular lesions with open wounds resulting from ruptured vesicles distributed along the right T10 dermatome.

A 64-year-old woman with a history of hypertension, asthma, and right-sided hemiplegia secondary to a hemorrhagic stroke sustained 10 years prior presented to the Infectious Diseases Department of our hospital with painful vesicular lesions over her right flank (T10 dermatome) that had developed 1 week earlier. She had previously been diagnosed with HZ at a local clinic and completed a 7-day course of oral famciclovir. However, due to persistent severe pain around the right flank and periumbilical region, along with open wounds resulting from ruptured vesicles, the patient was referred to our tertiary care center (Fig. 1).

At presentation, the patient reported a numerical rating scale (NRS) score of 8. Laboratory investigations revealed elevated levels of inflammatory markers, with an ESR of 54 mm/h and a CRP level of 9.50 mg/L. The white blood cell count was within normal limits at 6300/µL, and the neutrophil percentage was within the normal range.

Based on these findings, a diagnosis of secondary bacterial infection of the HZ lesions was established. A single intravenous dose of 2 g of ceftriaxone, a third-generation cephalosporin, was administered. Additionally, oral cefditoren (100 mg, three times daily [TID]) and acetaminophen (650 mg, TID) were prescribed for 1 week. The symptoms improved after antibiotic therapy and local wound care, with a reduction in the NRS score to 5 and partial resolution of the skin lesions.

**Figure 2.**
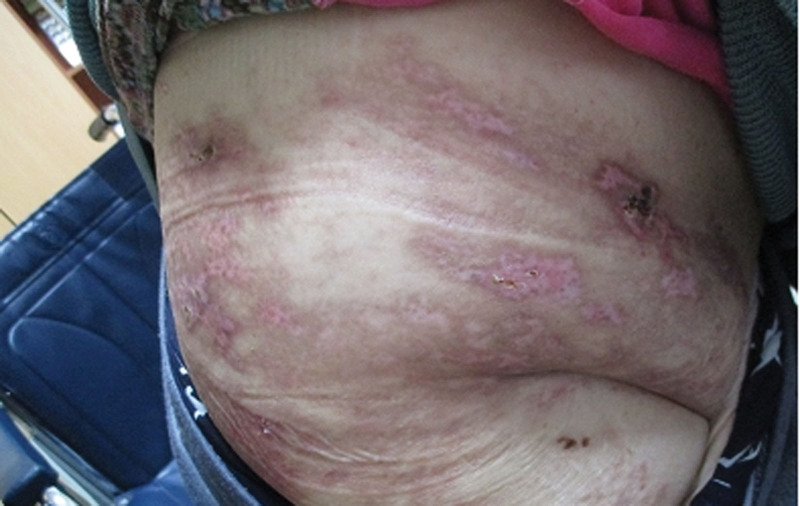
Photograph obtained after 3 weeks of hospitalization (4 weeks after herpes zoster onset), demonstrating marked improvement in the vesicular skin lesions.

However, 2 weeks later, approximately 4 weeks after the initial appearance of the rash, despite ongoing medical management and noticeable improvement in the skin lesions (Fig. 2), the patient reported worsening lower back and abdominal pain (NRS score, 8) accompanied by sleep disturbances. Subsequent laboratory investigations revealed markedly elevated CRP (88.8 mg/L) and ESR (65 mm/h). Although the white blood cell count remained within the normal range (6800/µL), the percentage of neutrophils increased to 77.5%.

The infectious disease team performed an abdominal ultrasound and computed tomography of the abdomen and pelvis to exclude intra-abdominal pathologies. No abnormalities were observed that could explain the clinical symptoms. Consequently, the patient was treated for zoster-associated pain using a combination of tramadol/acetaminophen, gabapentin, and amitriptyline.

In the fifth week after symptom onset, the patient was referred to a pain clinic because of persistent right flank and abdominal pain, with an NRS score of 8. During a detailed history, the patient reported pain that improved with rest in the supine position but worsened with spinal rotation and postural changes.

On physical examination, marked midline tenderness was noted over the lumbar spinous processes, and percussion elicited reproducible pain in the same region. Although the vesicular rash had resolved, inflammatory markers remained elevated, with a CRP level of 36.64 mg/L and an ESR of 60 mm/h. Given these findings, further evaluation was warranted, and a non-contrast MRI scan of the thoracolumbar spine, along with bone mineral density testing, was performed.

**Figure 3.**
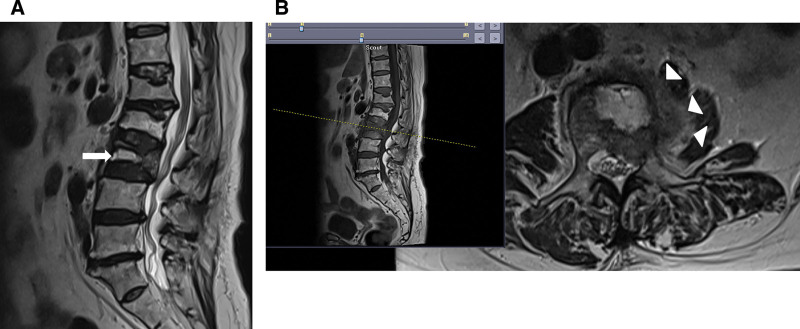
Non-contrast magnetic resonance imaging of the lumbar spine. (A) Sagittal T2-weighted image showing the collapse of the L3 vertebral body with an intravertebral fluid cleft (arrow). (B) Sagittal T1-weighted and axial T2-weighted images demonstrating paravertebral soft tissue edema accompanied by fluid collection (arrowheads).

A bone mineral density test revealed a T-score of ˗2.9 in the lumbar spine (L1–L4), indicative of osteoporosis. Spinal MRI revealed collapse of the L3 vertebral body with an intravertebral fluid cleft, consistent with Kummell’s disease (Fig. 3).

Additional findings included paravertebral soft tissue edema and fluid collection, exhibiting a low-signal intensity rim on T2-weighted images and central-to-high-signal intensity on T1-weighted images involving the left psoas muscle. These imaging features were suggestive of a superimposed infection with possible hematoma or abscess formation.

**Figure 4.**
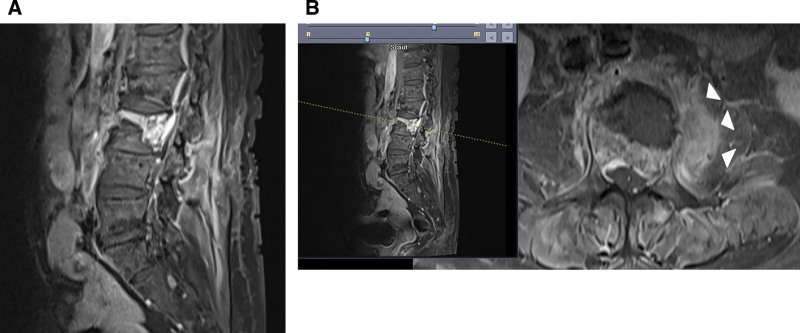
Contrast-enhanced magnetic resonance imaging of the lumbar spine. (A) Sagittal T1-weighted fat-saturated image showing the collapse of the L3 vertebral body with an intravertebral fluid cleft and associated bone marrow edema. (B) Axial T1-weighted fat-saturated image at the level of the L3 vertebra demonstrating paravertebral soft tissue enhancement (greater on the left than right) and mildly improved edematous swelling of the left psoas muscle with a decreased amount of rim-enhancing fluid collection (arrowheads).

Subsequently, contrast-enhanced MRI was performed to further evaluate the infectious spondylitis. The scan demonstrated marked enhancement of the paraspinal soft tissues, extending into the left psoas muscle (Fig. 4). Based on these findings, the radiology report indicated the differential diagnosis of infectious spondylitis with abscess formation or edema and hematoma related to a vertebral compression fracture.

Given the elevated ESR and CRP levels along with the MRI findings, an infectious disease team was consulted. To identify the causative pathogen, antibiotic therapy was withheld for 1 week, and a computed tomography-guided aspiration biopsy was performed. However, subsequent bacterial cultures, Gram staining, acid-fast bacilli staining and culture, *Mycobacterium tuberculosis* polymerase chain reaction, nontuberculous mycobacterial polymerase chain reaction, and histopathological examination yielded negative results.

Despite negative microbiological findings, the infectious disease team recommended initiating empirical treatment for presumed infectious spondylitis based on the clinical presentation and imaging characteristics. Cefazolin (2 g every 8 hours), a first-generation cephalosporin, was administered intravenously. At the initiation of antibiotic therapy, laboratory results showed a CRP level of 31.89 mg/L, an ESR of 69 mm/h, and a serum procalcitonin (PCT) level of 0.11 ng/mL.

Considering the potential contributions of the OVCF and HZ to the persistent pain reported by the patient, additional pharmacological interventions were initiated. Pregabalin (50 mg, twice daily) and amitriptyline (5 mg) were prescribed to treat the neuropathic pain associated with HZ. Oxycodone (5 mg twice daily) was initiated to manage mechanical pain secondary to OVCF. The patient was hospitalized, and intravenous antibiotic therapy was continued.

Laboratory parameters were assessed every 2 days following the initiation of intravenous antibiotic therapy. By day 6, both PCT and CRP levels had normalized (CRP < 5 mg/L; PCT < 0.5 ng/mL), and the ESR had decreased to 34 mm/h, representing a reduction of>50% from baseline.

Despite the normalization of inflammatory markers, the patient continued to experience significant movement-induced back pain, with only minimal discomfort reported at rest. No new neurological deficits were observed, apart from the preexisting right hemiplegia. These findings suggested that residual pain is more likely to be attributable to the OVCF than to an ongoing infection or HZ.

**Figure 5.**
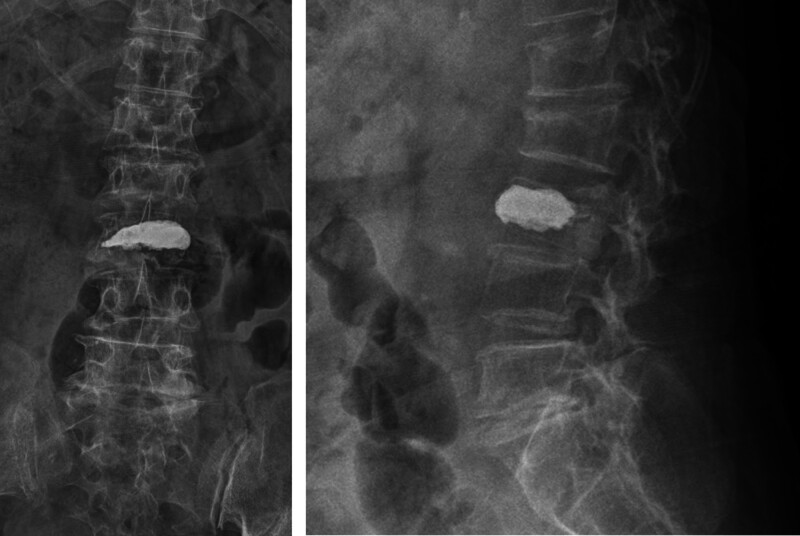
Post-vertebroplasty anteroposterior (AP) and lateral radiographs of the lumbar spine. (A) AP and (B) lateral views demonstrate adequate cement filling within the L3 vertebral body, with bilateral extension covering the posterior two-thirds of the vertebral structure.

In consultation with the infectious disease team, VP was deemed appropriate after a minimum of 2 weeks of intravenous antibiotic therapy, and the CRP and PCT levels had remained within normal limits. VP has been associated with improved mobility, reduced risk of pulmonary and gastrointestinal complications, and a relatively low incidence of post-procedural infectious exacerbation. The procedure was performed after informed consent was obtained from the patient and her family members. Post-procedural spinal radiographs (Fig. 5) revealed adequate cement filling within the L3 vertebral body, with bilateral distribution extending to the posterior two-thirds.

The patient reported substantial relief from back pain within 1 day of VP. Inflammatory markers, including CRP and PCT, remained within normal limits, with no evidence of recurrent infection.

Intravenous antibiotic therapy was continued for an additional 3 weeks, lasting for a total of 5 weeks, before transitioning to oral amoxicillin/clavulanate (625 mg TID). Oral antibiotic therapy was maintained for 2 months after discharge. At outpatient follow-up, the patient remained asymptomatic, with all laboratory parameters within normal limits. Therefore, the antibiotic therapy was discontinued. At discharge, the patient reported a substantial improvement in pain at the site of the HZ lesions. In the outpatient follow-up examination 3 months after discontinuing antibiotics, the pain remained stable, and the laboratory indicators remained within the normal range. Consequently, the medications prescribed for HZ were gradually tapered and discontinued (Table 1).

**Table 1:** Chronological summary of clinical events.

Time (from rash onset)	Key events
Week 1	Painful vesicular rash over right flank (T10); diagnosed with HZ at local clinic; 7-d oral famciclovir completed
Week 2	Persistent pain (NRS 8); elevated ESR/CRP; IV ceftriaxone + oral cefditoren given for secondary infection; partial skin improvement
Week 4	Worsening flank/abdominal pain; ESR/CRP markedly increased; presumed postherpetic neuralgia; started tramadol/acetaminophen, gabapentin, amitriptyline
Week 5	Referral to pain clinic; pain aggravated by posture/rotation; exam showed midline tenderness; MRI: L3 collapse (Kummell’s disease) + paravertebral/psoas involvement; osteoporosis confirmed (T-score -2.9)
Week 6	CT-guided aspiration biopsy (all cultures/PCR negative); empirical IV cefazolin started
Week 7	Inflammatory markers normalized after 6 d of IV therapy, but severe movement-induced pain persisted
Week 8	Vertebroplasty performed after ≥2 wk of IV antibiotics and normalization of CRP/PCT; immediate pain relief
Week 8–12	Continued IV antibiotics (total 5 wk) then switched to oral amoxicillin/clavulanate for 2 mo
Follow-up	Stable recovery, no recurrence of infection, marked pain improvement, HZ medications tapered and discontinued

CRP = C-reactive protein, CT = computed tomography, ESR = erythrocyte sedimentation rate, HZ = herpes zoster, IV = intravenous, MRI = magnetic resonance imaging, NRS = numerical rating scale, PCR = polymerase chain reaction, PCT = procalcitonin.

### 2.1. Patient perspective

**Figure 6.**
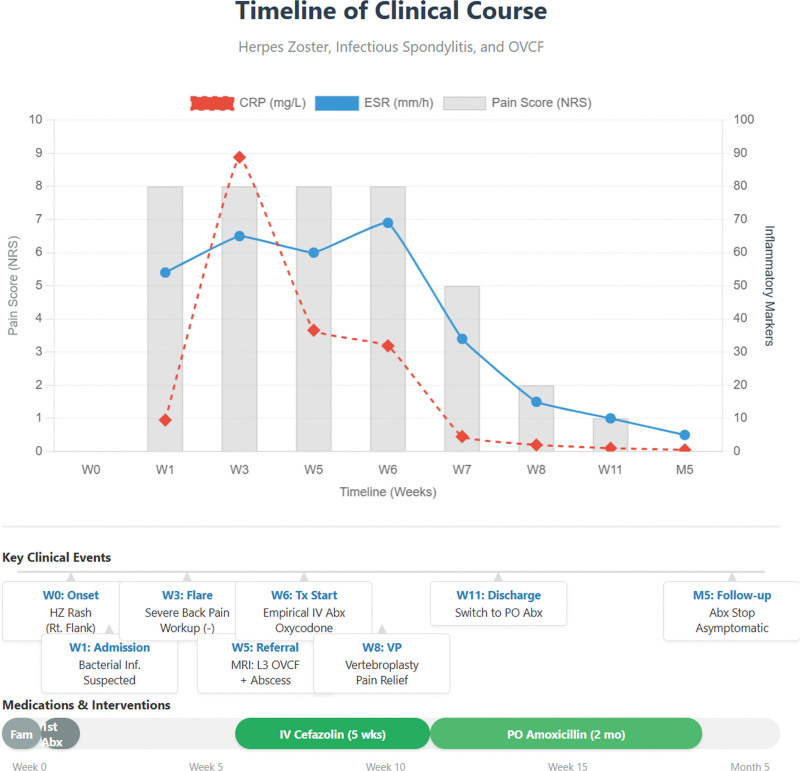
Timeline of the clinical course, laboratory findings, and interventions. The timeline illustrates the progression of symptoms and diagnostic marker levels over the treatment period. Despite initial antiviral treatment for herpes zoster, the patient experienced a worsening of symptoms and inflammatory marker expression. Following the diagnosis of osteoporotic vertebral compression fracture and infectious spondylitis, empirical antibiotic therapy led to the normalization of inflammatory markers. Vertebroplasty was performed after infection control, resulting in significant pain relief. Abx = antibiotics, CRP = C-reactive protein, ESR = erythrocyte sedimentation rate, IV = intravenous, MRI = magnetic resonance imaging, NRS = numerical rating scale, OVCF = osteoporotic vertebral compression fracture, PO = per Os, VP = vertebroplasty.

The patient initially believed that their pain was solely due to HZ and expressed frustration when symptoms persisted despite antiviral therapy. They reported significant anxiety and sleep disturbances during the diagnostic process, particularly when laboratory results remained abnormal. Following VP, the patient reported rapid pain relief and improved mobility, which were considered turning points in their recovery. They expressed gratitude for the thorough evaluation that ultimately clarified the coexistence of HZ, OVCF, and infectious spondylitis and stated that regaining the ability to rest comfortably was the most meaningful aspect of treatment (Fig. 6).

## 3. Discussion

This case underscores the essential role of physical examination in identifying coexisting pathologies even after an initial diagnosis has been established. Although the patient presented with a unilateral vesicular rash along the dermatome suggestive of HZ, the exacerbation of pain with movement, deviation from the expected clinical progression, and rising levels of inflammatory markers necessitated further diagnostic evaluation. Additional physical examinations and imaging revealed concomitant OVCF and infectious spondylitis. This expanded diagnostic approach resulted in significant modifications to the treatment plan, ultimately improving clinical outcomes.

Physical examination is a fundamental component of clinical assessment and diagnosis. Although advances in imaging and laboratory testing have reduced the emphasis on physical examination in modern practice, it remains a rapid and direct tool for assessing a patient’s condition. Even after a definitive diagnosis consistent with clinical findings has been established, comprehensive history-taking and physical examination remain essential to identify additional underlying pathologies and ensure diagnostic accuracy.^[14]^

HZ is typically diagnosed based on its distinctive clinical presentation, including dermatomal vesicular eruptions and localized pain.^[7]^ However, when atypical findings are observed on physical examination or laboratory testing, clinicians should consider alternative diagnoses or complications and perform additional imaging studies as appropriate.

In the current case, the patient was diagnosed with concurrent OVCF and infectious spondylitis complicated by a paraspinal abscess, in addition to HZ. Pyogenic spondylitis following a vertebral compression fracture is exceedingly rare, with only 14 cases reported in the literature to date.^[15]^ Most recently, 5 cases were reported in which the pathogen was identified after OVCF, and the average time from OVCF to diagnosis was 37 days.^[16]^ The development of pyogenic spondylitis following a compression fracture typically requires the presence of vertebral injury and concurrent bacteremia.

Infectious spondylitis may have developed approximately 3 weeks after symptom onset, coinciding with the initial improvement in zoster-associated pain, followed by sudden exacerbation of back pain and a marked elevation in CRP levels. This assumption is supported by the 2015 Clinical Practice Guidelines of the Infectious Diseases Society of America, which recommend considering the diagnosis of spinal infection in patients presenting with new or worsening localized spinal pain accompanied by elevated ESR or CRP levels.^[17]^ However, the exact timing of infectious spondylitis involving compressed vertebrae remains uncertain. The patient reported no trauma preceding or following the onset of HZ, and no imaging data were available. Thus, the precise etiology and timing of the infection could not be definitively established.

Although the simultaneous occurrence of HZ, OVCF, and infectious spondylitis may initially appear to be a diagnostic coincidence, a potential pathophysiological cascade linking these conditions warrants consideration. First, the reactivation of varicella–zoster virus itself is a clinical marker of reduced cell-mediated immunity, suggesting that the patient may have had a baseline vulnerability to infection.^[18]^ Furthermore, the physiological stress induced by severe pain from both the acute HZ and the OVCF can stimulate the hypothalamic–pituitary–adrenal axis. The resulting release of endogenous corticosteroids induces a transient state of systemic immunosuppression, potentially increasing susceptibility to secondary bacterial infections.^[19]^

In addition to systemic factors, the OVCF site likely acted as a locus minoris resistentiae (a place of less resistance). The disruption of local vasculature and the formation of a fracture hematoma provide an ideal culture medium for bacteria.^[20]^ Therefore, it is plausible that transient bacteremia **–** possibly originating from a minor skin breach associated with HZ or another occult source **–** seeded the vulnerable fracture site, leading to the development of infectious spondylitis in this immunocompromised patient.

In this context, the cutaneous HZ lesions likely served as the primary portal of entry for the pathogen. The patient presented with open wounds from ruptured vesicles and was diagnosed with a secondary bacterial infection at their initial visit. We hypothesized that this breach in the skin barrier facilitated transient bacteremia. Crucially, the concurrent OVCF site acted as a vulnerable target for hematogenous seeding. The fractured vertebra, particularly with the intravertebral vacuum cleft indicative of Kummell’s disease, was characterized by a disrupted vascular supply and localized necrosis. This ischemic environment, combined with the presence of a fracture hematoma, created a locus minoris resistentiae, a site of lowered resistance, that was highly susceptible to colonization by circulating bacteria, even in the absence of direct external trauma to the spine.^[20,21]^ This hypothesis-generating observation underscores the need for heightened clinical vigilance and further investigation in similar clinical contexts.

Imaging modalities, such as MRI and bone scintigraphy, are essential for diagnosing acute OVCFs and infectious spondylitis.^[5,12]^ However, physical examination findings also contribute significantly to diagnostic accuracy.^[3,12]^ In patients with OVCFs, physical examination typically reveals midline spinal tenderness and accentuated kyphosis upon percussion of the affected vertebrae.^[3]^ Patients may report worsening pain during spinal rotation or with changes in posture.^[3]^ Langdon et al^[12]^ evaluated the diagnostic performance of these 2 signs and reported that the percussion sign had a sensitivity of 87.5% and specificity of 90%, whereas the supine pain sign, defined as pain exacerbation preventing the patient from lying flat, showed a sensitivity of 81.25% and specificity of 93.33%.

In the present case, the physical examination at the time of presentation to the pain clinic revealed clear signs of spinal pain aggravated by movement and midline tenderness elicited by percussion. These clinical findings prompted further evaluation of alternative pain etiologies beyond HZ.

Persistent back pain unrelieved by rest, particularly when accompanied by fever or elevated levels of inflammatory markers, suggests the possibility of infectious spondylitis.^[13]^ Although localized spinal pain occurs in over 90% of infectious spondylitis cases, fever is reported in only approximately 50% of cases.^[5]^ In the current case, localized back pain, elevated ESR, and increased CRP levels were observed; however, the absence of fever rendered the diagnosis of infectious spondylitis less apparent prior to MRI.

Specifically, we suspected a spinal compression fracture based on physical examination and MRI findings. Based on the marked enhancement of the paraspinal soft tissues on MRI, we suspected infectious spondylitis. Furthermore, the patient had no specific neoplastic disease, and imaging analysis revealed no tumor-related findings, thus ruling out the possibility of metastatic spinal disease.

Unlike ESR and CRP levels, which are nonspecific markers of inflammation, serum PCT levels are useful in distinguishing bacterial infections. However, in this case, PCT levels were not assessed until after MRI, thereby limiting their utility in facilitating the early diagnosis of infectious spondylitis.^[22]^ Neurological deficits may assist in diagnosing infectious spondylitis.^[5]^ However, the patient in the current case had preexisting right-sided hemiplegia secondary to a hemorrhagic stroke sustained 10 years prior and exhibited no new neurological deficits during the clinical course.

The initial management of OVCFs typically involves conservative measures such as analgesia, spinal orthosis application, and modification of physical activity. However, if severe pain or functional limitations persist despite conservative therapy, interventional procedures such as VP may be considered.^[3,23,24]^ In contrast, prompt initiation of antibiotic therapy is essential after the diagnosis of infectious spondylitis. In certain cases, surgical intervention, including incision and drainage, may be necessary to achieve infection control.^[5,13]^

VP and KP are contraindicated in patients with active spinal infection,^[25,26]^ because performing these procedures in an infected field may exacerbate the infection or its dissemination. Therefore, eradication of the infection must be prioritized, and the decision to perform any spinal intervention should be made after careful consideration of timing and clinical necessity. However, specific clinical guidelines and robust studies on the optimal timing of VP and KP interventions for concurrent spinal infections are lacking. Most of the available literature addresses the efficacy and complications of these procedures in noninfectious OVCFs.^[27]^

The most controversial aspect of this case was the decision to perform VP in a recently infected spinal field. While specific clinical guidelines for this scenario are lacking, data regarding the safety of VP in “treated” or “quiescent” spondylitis are accumulating. Studies have reported that VP can be safely performed in healed or chemically treated cases of spinal tuberculosis without reactivating the infection in patients whose inflammatory markers have normalized.^[28]^ Although evidence specific to pyogenic spondylitis is limited, the principle of relying on the normalization of CRP and ESR as a surrogate marker for a “clinical cure” suggests that once the active inflammatory phase has resolved, the risk of procedure-related exacerbation is significantly mitigated.^[17]^ Based on this rationale, a calculated risk–benefit assessment was undertaken, and we proceeded with VP to address the persistent mechanical pain.

In this case, the decision to proceed with VP after 2 weeks of intravenous antibiotic therapy was based on multiple predefined criteria indicating that the infection had stabilized rather than being based on the duration of antibiotic therapy alone. First, inflammatory biomarkers demonstrated a favorable and sustained response to antimicrobial treatment, with normalization of both CRP and PCT levels and a >50% reduction in ESR, which has been associated with effective infection control in spinal infections.^[29]^ Second, the patient exhibited no systemic signs of an ongoing infection, such as fever or hemodynamic instability, and no new or progressive neurological deficits were observed. Third, the patient’s pain phenotype evolved during treatment: pain at rest was well controlled, whereas severe movement-induced pain persisted, suggesting a predominantly mechanical origin attributable to OVCF rather than active infection. Finally, prolonged immobilization due to uncontrolled mechanical pain posed substantial risks to this elderly patient, including deconditioning, pulmonary complications, and delayed rehabilitation.^[30,31]^

Although conventional recommendations often suggest a 4 to 6-week course of intravenous antibiotics before considering spinal interventions, such time-based thresholds are largely empirical and not supported by high-quality evidence, particularly in patients with concomitant OVCFs.^[20,32]^ In the present case, VP was undertaken as a calculated risk under close supervision by a multidisciplinary team after multiple objective indicators of infection stabilization had been met. The absence of post-procedural clinical deterioration or rebound elevation of inflammatory markers supports the validity of this individualized, response-based decision-making approach. Importantly, this case does not advocate early VP as a standard strategy; rather, it suggests that the optimal timing and safety of vertebral augmentation in patients with active or recently treated spinal infections should be determined on a case-by-case basis using a structured, criteria-driven framework and multidisciplinary clinical judgment.

In the present case, although the patient was diagnosed with infectious spondylitis and treated with antibiotics, an image-guided biopsy yielded negative results. Previous studies have indicated that when a spinal infection is clinically suspected but initial cultures are negative, a second biopsy may enhance the diagnostic yield.^[18,33,34]^ However, a second biopsy was not performed in this case, as empirical antibiotic therapy was initiated immediately, potentially reducing the likelihood of subsequent pathogen isolation from culture specimens.^[35]^

The patient had received oral antibiotics for 1 week before the first biopsy that was prescribed for a presumed secondary bacterial skin infection associated with HZ. Despite the discontinuation of antibiotics prior to biopsy, culture sensitivity may have remained low.^[35]^ Therefore, considering the clinical and imaging findings and the limited diagnostic utility of a second biopsy, empirical intravenous antibiotic therapy was promptly initiated.

This case is unique in that it illustrates the coexistence of 3 distinct pathologies, HZ, OVCF, and infectious spondylitis, in a single older patient, a combination that has rarely been described in the literature. The simultaneous occurrence of these conditions created significant diagnostic complexity and posed therapeutic challenges. Furthermore, the decision to proceed with VP while the infection was still being managed highlighted the need to carefully balance the risks of exacerbating the infection with the need to stabilize the fracture and relieve pain. This rare overlap and favorable outcome following a multidisciplinary approach underscores the clinical value of individualized decision-making in such complex scenarios.

This study has certain limitations. After consultation with the infectious disease team, VP was performed with informed consent from the patient and guardian, based on a risk–benefit analysis. Following sustained improvement in inflammatory markers after a minimum of 2 weeks of antibiotic therapy, favorable outcomes were achieved. Although a computed tomography-guided biopsy was performed, all cultures and molecular tests were negative, which limited the definitive confirmation of infectious spondylitis. Moreover, no established clinical studies have reported criteria for spinal intervention in the presence of infectious spondylitis. Therefore, a multidisciplinary randomized controlled clinical trial is warranted to establish the criteria for spinal intervention in infectious spondylitis and guide clinical decision-making.

## 4. Conclusion

This case highlights the importance of integrating thorough history-taking and physical examination with laboratory and imaging data to achieve an accurate differential diagnosis. Furthermore, this case demonstrates that VP can be safely performed in patients with suspected infectious spondylitis when objective evidence of reduced inflammation is present after a minimum of 2 weeks of antibiotic therapy. To the best of our knowledge, this is the first reported case describing the coexistence of HZ, OVCF, and infectious spondylitis, managed successfully with VP during ongoing infection control. In elderly patients presenting with HZ and persistent or worsening back pain, clinicians should maintain a high index of suspicion for concomitant OVCF and spinal infection. Early recognition and a multidisciplinary approach encompassing infection control, fracture stabilization, and management of HZ are essential for optimizing outcomes. This report underscores the necessity for individualized evidence-based therapeutic strategies for the management of complex spinal infections and reinforces the value of collaborative multidisciplinary care to achieve optimal patient outcomes.

## Acknowledgments

We would like to thank Editage (www.editage.co.kr) for the English language editing.

## Author contributions

**Conceptualization:** Chung Hun Lee.

**Data curation:** Chung Hun Lee.

**Investigation:** Jaeeun Lee, Chung Hun Lee.

**Methodology:** Jaeeun Lee.

**Project administration:** Chung Hun Lee.

**Resources:** Chung Hun Lee.

**Software:** Jaeeun Lee.

**Supervision:** Chung Hun Lee.

**Validation:** Jaeeun Lee, Chung Hun Lee.

**Visualization:** Chung Hun Lee.

**Writing – original draft:** Jaeeun Lee, Chung Hun Lee.

**Writing – review & editing:** Chung Hun Lee.
